# An Unusual Case of Peripheral Nerve Vasculitis

**DOI:** 10.1155/2024/3469182

**Published:** 2024-01-29

**Authors:** S. Wang, Arsany A., D. Feinstein, P. Traisak, H. Eid, M. Karpoff

**Affiliations:** ^1^Cooper University Health Care, Camden, NJ, USA; ^2^Care Point Health, Jersey City, NJ, USA; ^3^Cooper Medical School of Rowan University, Camden, NJ 08103, USA

## Abstract

Peripheral neuropathy is a common manifestation of systemic vasculitis. The etiology of vasculitic peripheral neuropathy is generally classified into two groups: systemic and nonsystemic. In systemic vasculitic neuropathy (SVN), neuropathy is a consequence of a systemic disease, most commonly involving medium and small vessels throughout the body. There are three main clinical presentations: multifocal neuropathy, distal symmetric polyneuropathy, and overlapping multifocal neuropathy. Specifically, distal symmetric polyneuropathy affects multiple somatic nerves diffusely in a symmetric and length-dependent pattern (also known as the classic stocking-glove pattern). This case represents an atypical presentation of SVN, presenting with widespread symmetric polyneuropathy.A 73-year-old woman presented with distal acute on chronic bilateral upper and lower extremity weakness, sensory changes, and widespread pain. Symptoms started about three months prior and gradually worsened with progressive difficulty with ambulation and required assistive devices. Elevated ESR is at 70 mm/hour, CRP at 25.66 mg/dL, elevated c-ANCA titers at 1 : 320 and PR3 at 5.0 AI, and elevated creatine kinase (CK) at 500–600 U/L. A muscle biopsy of the left vastus showed neurogenic atrophy without myositis. Initial improvement was with oral prednisone, but was stopped on discharge. Many purpuric and petechial lesions were developed on distal legs/feet and right fourth digit distal gangrene. EMG showed distal, symmetric, and axonal polyneuropathy affecting the upper and lower extremities and acute denervation in more distal muscles. The patient received pulse dose steroids and two doses of rituximab induction therapy and was discharged with an oral steroid taper. The patient's symptoms started as distal symmetric neuropathy at the onset and progressively worsened over the course of 3 months. Neuropathy, both on the exam and on EMG, seemed to have developed more rapidly than expected, regardless of its distribution. The EMG showed severe peripheral nerve damage and denervation, which is unusual for ANCA-associated systemic vasculitis.

## 1. Introduction

Peripheral neuropathy is commonly present in vasculitis; it is caused by inflammation and ischemic injury to the vasa nervosum which supplies blood to peripheral nerves [1]. Etiology of peripheral neuropathy in vasculitis is generally classified into two groups: systemic and nonsystemic vasculitis neuropathy. In systemic vasculitis neuropathy (SVN), neuropathy is a consequence of systemic disease, most commonly involving medium and small vessels throughout the body; this can be a primary process (such as in antineutrophil cytoplasmic autoantibody- (ANCA-) associated vasculitis or polyarteritis nodosa (PAN)) or a secondary process (such as in vasculitis associated with rheumatoid arthritis (RA), systemic lupus erythematosus (SLE), or Sjogren's syndrome) [1–3]. In nonsystemic vasculitis neuropathy (NSVN), injury is limited to just blood vessels that supply the peripheral nervous system and occurs much less commonly than systemic vasculitis as a cause of peripheral neuropathy (∼10%) [1–3]. There are three main patterns of clinical presentation of peripheral neuropathy from vasculitis: multifocal neuropathy, overlapping multifocal neuropathy, and distal symmetric polyneuropathy. Multifocal neuropathy (also known as mononeuropathy multiplex) affects two or more noncontiguous somatic, sensory, motor, or sensorimotor peripheral or cranial nerves simultaneously or sequentially. Distal symmetric polyneuropathy affects multiple somatic nerves diffusely in a symmetric and length-dependent pattern. Overlapping multifocal neuropathy is a combination of the two previous patterns [1, 3].

## 2. Case

A 73-year-old female presented with a medical history of hyperlipidemia and treated tuberculosis in 2010. She initially presented with acute on chronic upper and lower extremity weakness bilaterally, sensory changes, as well as diffuse neuropathic pain as transferred from an outside hospital. Symptoms started about three months prior and gradually worsened; the patient was in good health prior. Progression of symptoms was in a symmetric manner and eventually led to difficulties with ambulation, and the patient required assistive devices such as cane and wheelchair. When initially assessed, the patient had 2/5 strength on the left side and 3/5 on the right side in the upper and lower extremities bilaterally. Sensory changes primarily were numbness and tingling at fingertips and toes extending into the hands and feet and also had poor temperature sensation in feet, which led to a burn injury on the sole of the right foot. Neuropathic pain was severe, widespread, and diffuse; this was the patient and family's primary concern, described as a constant burning sensation that was worst in the hands and feet. Patient was initially diagnosed with spinal stenosis at an outside hospital on prior admission and was given corticosteroid injections in spine, pregabalin, and duloxetine which were all ineffective at controlling symptoms.

On initial admission, symptoms were initially thought to be from inflammatory myositis, and prednisone 40 mg daily was empirically started, and MRI left thigh and muscle biopsy were obtained. MRI left thigh showed multifocal patchy/nodular intramuscular edema throughout the muscles of the pelvis and bilateral thighs; a muscle biopsy showed neurogenic atrophy with no myositis. Other results showed elevated ESR at 70 mm/hour, elevated CRP at 25.66 mg/dL, elevated c-ANCA titers at 1 : 320 (normal < 1 : 20), elevated PR3 antibody at 5.0 AI (normal < 1.0), urine microalbumin to creatinine ratio was 88.7 *μ*g/mg and urinalysis showed 2+ protein, 1+ glucose, 3+ blood, and 3+ leukocyte esterase, normal complement 3 and 4 levels, elevated creatine kinase (CK) in 500–600 U/L (normal 0–200 U/L), normal MRI of cervical spine, MRI brain showed pachymeningeal enhancements, computed tomography of chest showed multifocal ground-glass opacities and mild diffuse interlobular septal thickening, compatible with pulmonary interstitial edema, lumbar puncture with elevated RBC at 22 thousand/*μ*L, cell count at 43/*μ*L, glucose at 93 mg/dL, and proteins were normal (ultimately deemed as noninflammatory). The patient had mild improvement on prednisone 40 mg daily with a return of muscle strength to 3/5 on the left side and 4/5 on the right side and the patient was eventually discharged to a rehabilitation facility. There were concerns for ANCA-associated vasculitis; thus, prednisone was continued upon discharge along with prophylactic trimethoprim-sulfamethoxazole three times weekly, with close follow-up with outpatient rheumatology arranged. Electromyography (EMG) could not be obtained inpatient; thus, outpatient EMG was arranged.

Unfortunately, prednisone was stopped a few days after discharge due to elevations in blood glucose, and patient's symptoms of muscle weakness and neuropathic pain worsened, to the point that the patient was bed-bound and needed assistance with feeding and all other activities of daily living. Follow up appointment through telemedicine showed new severe diffuse muscle wasting, petechiae on toes and fingertips diffusely, as well as right 4th digit distal phalanx gangrene and oral ulcers, and the patient was readmitted shortly afterwards.

On second admission, the patient was given pulse dose steroids (250 mg solumedrol daily for three days), followed by oral prednisone taper. When initially assessed, there was severe muscle weakness at 1/5 on the left and 2/5 on the right, along with petechiae diffusely on the lower extremities, toes, and fingertips bilaterally. Patient responded well to prednisone with a significant improvement of the muscle strength to 3/5 on the left and 4/5 on the right and resolution of petechiae. Of note, CK normalized to 38 U/L. EMG was performed in patient that showed peripheral nerve vasculitis which was severe and in a symmetric pattern in the upper and lower extremities, predominantly with axon loss. A nerve biopsy was considered, but based on EMG results showing extensive nerve damage, it was unclear if biospy would have high yield and thus not performed. Given development of new symptoms as well as positive c-ANCA titers and PR3 antibodies, the patient was diagnosed with ANCA-associated vasculitis and started rituximab induction therapy. Patient received first two doses of rituximab induction therapy inpatient and was discharged due to improvement of symptoms along with oral steroid taper. Pt was started on insulin due to development of steroid-induced hyperglycemia during the second admission. Shortly after discharge, patient became hypoglycemic at home and became unresponsive; she expired after never recovering from the hypoglycemia.

## 3. Discussion

SVN generally presents peripheral neuropathy in an asymmetric pattern, thought to be due to systemic vasculitis affecting nerve blood supply in a patchy pattern. NSVN generally presents as distal and symmetric sensorimotor polyneuropathy, thought to be due to vasculitis being limited to blood vessels supplying peripheral nerves. The above case represents an atypical presentation of SVN presenting with distal symmetric polyneuropathy [4].

In ANCA-associated vasculitis, peripheral neuropathy presents in variable rates (granulomatosis with polyangiitis 15–25%, eosinophilic granulomatosis with polyangiitis 60–70%, and microscopic polyangiitis 40–50%), most commonly presents in multifocal neuropathy (classically as mononeuritis multiplex) with sensory, motor, or sensorimotor deficits in an asymmetric pattern [4]. Symmetric polyneuropathy can present as a late manifestation of ANCA vasculitis, generally in patients with untreated disease for greater than three months, likely due to the accumulation of peripheral nerve damage [5, 6]. Our patient's symptoms started as distal symmetric neuropathy at the onset and progressively worsened in distal symmetric pattern over the course of 3 months. EMG was obtained about three months into the disease course and already showed severe peripheral nerve damage, but systemic manifestations of ANCA vasculitis only manifested around that same time, suggesting that most of the vasculitis injury was accumulated in peripheral nerves, which is also unusual for ANCA-associated vasculitis.

Drug-induced peripheral neuropathy was also considered, as the patient had two close hospitalizations as well as treatment with many different types of medications, including immunosuppressive agents and antibacterials. Of the medications which patient was started on are rituximab, cefepime, vancomycin, trimethoprim-sulfamethoxazole, and ceftriaxone, and none are classically associated with peripheral neuropathy [7]. The patient was never started on tumor necrosis factor alpha (TNF-alpha) inhibitors, leflunomide, and treatment for tuberculosis such as isoniazid, ethambutol, or linezolid which have all be associated with higher rates of peripheral neuropathy [7].

## 4. Conclusion

We believe the above case represents an unusual presentation of acute distal symmetric pattern of peripheral neuropathy in ANCA-associated vasculitis. While rare, it is important to recognize and associate this pattern of peripheral neuropathy with SVN, as it can present prior to other systemic or organ-specific signs and symptoms [8]. Timely initiation of treatment is important to avoid the progression of disease and additional morbidity and mortality.
